# Clinical, Serological, and Immunological Characteristics of Greek Patients with Polymyalgia Rheumatica and/or Giant Cell Arteritis: A Research Protocol

**DOI:** 10.31138/mjr.050923.csa

**Published:** 2023-09-05

**Authors:** Tereza Memi, Nikolaos Koletsos, Nafsika Gerolymatou, Maria Karakosta, Athanasios N. Georgiadis, Alexandros A. Drosos, Paraskevi V. Voulgari

**Affiliations:** Rheumatology Clinic, Department of Internal Medicine, Medical School, University of Ioannina, Ioannina, Greece

**Keywords:** polymyalgia rheumatica, giant cell arteritis, biomarkers, pentraxin, osteopontin, calprotectin

## Abstract

Polymyalgia Rheumatica (PMR) and Giant Cell Arteritis (GCA) are chronic inflammatory disorders that usually affect older people. Although the aetiology of these diseases remains unknown, genetic, environmental, and immune factors have been implicated. Specific cytokines such as the IL-6, IL-1β, IL-12, IL-17, and interferon –γ seem to play an essential role. The diagnosis of the disease is usually based on clinical manifestations and the use of histology or imaging, while disease monitoring is based on physical examination, laboratory, and imaging findings. However, there is the unmet need in identifying possible biomarkers that could help the diagnosis and the monitoring as well. The present study aims to investigate the epidemiological, clinical, and immunological characteristics of PMR and/or GCA patients in the region of northwest Greece and to evaluate the role of specific molecules associated with the pathogenesis of the diseases, giving evidence to possible future biomarkers.

## INTRODUCTION

Polymyalgia Rheumatica (PMR) and Giant Cell Arteritis (GCA) are chronic inflammatory disorders of unknown origin, that usually affect people older than 50 years old, with peak incidence during the eighth decade of life (70–79 years).^1–4^ PMR is more common than GCA (about 3 times) and, although they can appear separately, PMR and GCA often overlap and they are considered to be part of the same disease spectrum.^4–6^ Thus, PMR is observed in about half (40–60%) of patients with GCA and, on the other hand, up to 21% of patients presenting with PMR manifestations have biopsy proven GCA.^3,4,7^ Both diseases are slightly more common in women (lifetime risk for PMR and GCA 2,4% and 1% respectively) than men (1,7 and 0,5% for PMR and GCA respectively). The highest incidence rates have been reported in Northern European countries (especially in the Scandinavian Peninsula) and in individuals of Scandinavian descent.^1,3,4,8^

The characteristic manifestations of PMR include pain and stiffness mainly of the shoulder girdle as well as of the pelvic girdle. Patients may, also complain of pain in the cervical region, upper arms, hips and thighs.^1,3^ GCA may present with the typical cranial symptoms (headache, scalp tenderness, temporal artery abnormalities on examination, jaw or tongue claudication, or visual symptoms, with permanent visual loss being the most serious and feared complication), PMR symptoms, constitutional symptoms (eg, anorexia, malaise, weight loss, fever) or symptoms of large artery involvement (eg, arm claudication or Raynaud’s phenomenon).^1,7,9,10^ The clinical features are, usually, accompanied by elevated inflammatory markers like erythrocyte sedimentation rate (ESR) and c-reactive protein (CRP) which have been included in the classification criteria.^3,9,11,12^ Often, anaemia and thrombocytosis are also present.^3,9^

The aetiology of PMR and GCA remain unknown. Both human leucocyte antigen (HLA) and non-HLA genetic risk factors have been implicated.^3,7^ A repeated association between HLA-DRB1 alleles (especially HLADRB1*04) and GCA susceptibility has been reported, while results regarding PMR remain controversial among different populations.^7,13,14^ It seems that HLA-DRB1 alleles may also be a risk factor for disease severity (eg, glucocorticoid resistance or risk for relapse).^15,16^ Other gene polymorphisms associated with PMR and/or GCA such as variants within the loci of interleukin-6, intercellular adhesion molecule or vascular endothelial growth factor have also been described.^3,13,14,17^ Besides genetic factors, environmental factors (eg, infections) have also been implicated in the pathogenesis of PMR and GCA.^3,14^ The inflammatory infiltrate in GCA is composed of CD4+ T lymphocytes, macrophages, and giant cells. Two major and separate immune-response networks participate in the vascular inflammation: Firstly, the IL-6/IL-1β/IL-17 axis. The IL-6 early in the disease, stimulates the differentiation of T lymphocytes to Th17 cells, which produce numerous cytokines that regulate local and systemic inflammatory responses (eg, increase of acute phase reactants, anaemia, thrombocytosis).^1,18^ Secondly, the IL-12/interferon –γ (IFN-γ) axis. The IL-12 induces Th1 cells to produce TNFα and IFN-γ. The latter activates macrophages, endothelial cells, vascular smooth muscle cells, fibroblasts leading to lumen–obstructive intimal hyperplasia (late manifestations).^1,18^ Proteolytic enzymes (eg, matrix metalloproteinases) and growth factors (vascular endothelial growth factor) promote remodelling of the arterial wall.^1^

The diagnosis of the disease is usually based on clinical manifestations and reinforced by the use of histology (temporal artery biopsy) or imaging (ultrasound, magnetic resonance angiography-MRA, PET scanning).^7^ The estimated sensitivity of temporal artery biopsy was calculated to be 77,3%.^19^ In individuals with ocular symptoms, however, positive temporal artery biopsies may be found up to 83%.^20^ Although disease monitoring is based on physical examination, laboratory findings (high ESR, CRP) and imaging, to date, there is the unmet need in the identification of possible biomarkers that could help not only the diagnosis of the disease but also the monitoring as well. There are promising results about serum Pentraxine-3 (PTX3), Osteopontin (OPN), Calprotectin as possible disease biomarkers.^21–28^

The purpose of this study is i) to investigate the epidemiological, clinical, serological, and immunological characteristics of PMR and/or GCA patients in Northwest (NW) Greece, ii) to evaluate serological and immunological parameters associated with the pathogenesis of the diseases and iii) to identify molecules as potential bio-markers that might have a prognostic value.

## MATERIALS AND METHODS

### Study Design

The study will use a cross-sectional design. Patients meeting the diagnosis of PMR (according to the 2012 EULAR/ACR classification criteria) and/or GCA (according to the 2022 EULAR/ACR classification criteria) will be prospectively recruited.^11,12^ Patients with a new diagnosis (before treatment initiation), along with patients having a flare (but before adjusting the treatment) will be included. The control group will include patients having a stable disease activity and treatment. The study was approved by the institutional review board committee and will be conducted in accordance with the Declaration of Helsinki (2013 revision).^29^ All patients will sign a consent form prior to participating. Patients with other autoimmune rheumatic diseases, active malignancies or infections will be excluded from the study.

After obtaining a complete medical history, clinical and demographic characteristic will be recorded. All patients will undergo routine laboratory testing (**Table 1**) including biochemical profile (eg, glucose levels, renal and liver function tests, electrolytes), inflammatory markers (ESR, CRP), and immunological testing (rheumatoid factor, anti-cyclic citrullinated peptide antibodies, antinuclear antibodies). Additionally, serum samples from all patients will be separated and stored at -80oC. Serum levels of specific interleukins (IL-1β/-6/-12/-17/-23), along with levels of IFN-γ, PTX-3, OPN and Calprotectin will be measured, according to a standard methodology. All the above molecules will be accurate detected by employing the quantitative sandwich enzyme immuno-assay technique. Commercially available competitive enzyme-linked immunosorbent assay kits (MyBioSource) will be used, and all samples will be analysed by the same investigator. The findings of these tests will be examined for possible associations with clinical findings, serological, and immunological parameters.

**Table 1.** Measured laboratory parameters of the study population.

1 Full blood count
2 Erythrocyte sedimentation rate
3 C-reactive protein
4 Glucose
5 Renal function tests (Urea, Creatinine)
6 Liver function tests (AST, ALT, γGT, ALP)
7 Creatine phosphokinase
8 Electrolytes (K^+^, Na^+^, Ca^++^, Mg^++^)
9 Immunological profile (RF, ANA, anti-CCP)
10 Interleukins (IL-1β, IL-6, IL-12, IL -17, IL-23)
11 Interferon-γ
12 Pentraxine -3
13 Osteopontin
14 Urinanalysis

AST: aspartate aminotransferase; ALT: alanine amino-transferase; γ-GT: γ-glutamyltransferase; ALP: alkaline phosphatase; K^+^: Potassium; Na^+^: Sodium; Ca^++^: Calcium; Mg^++^: Magnesium; RF: Rheumatoid factor; ANA: Antinuclear antibodies; anti-CCP: Anti-cyclic citrullinated peptide antibodies.

### Statistical Analysis

Statistical analyses will be performed using Microsoft Excel 2017 and SPSS software (IBM SPSS Statistics 25.0, Chicago, IL, USA). Continuous variables will be described as mean ± standard deviation or as median ± interquartile range, based on the normality of the distribution. Differences among groups will be examined by independent samples t-tests for normally distributed variables, while non-parametric Mann-Whitney test will be used for non-normally distributed variables. Qualitative variables will be compared by the χ^2^ test or Fisher’s exact test when necessary and results will be expressed as percentages. Pearson’s and Spearman’s correlations will be used for the univariate analysis, based on the variable’s normality of distribution. Depending on the cohort’s data, a multivariate analysis will be performed to identify independent risk factors for each of the variables.

## CONCLUSION

This is the first study in Greece trying to provide additional data on the characteristics of PMR and/or GCA patients in NW Greece. The results will possibly shed light on associations between clinical and serological markers. Moreover, the study aims to possibly identify new biomarkers that may have a prognostic value.

## STUDY APPROVAL

The study has been approved by the Ethics Committee of the University Hospital of Ioannina.
